# Does English proficiency matter? Testing its moderating role in the TAM for AI-enhanced MOOC adoption in vocational education

**DOI:** 10.3389/fpsyg.2026.1772129

**Published:** 2026-02-19

**Authors:** Shuhua Hou, Xianhe Liu, Xiaoqing Shen, Chenyun Zhang, Dali Liu, Yang Wu

**Affiliations:** 1 School of Higher Vocational Education, The Open University of Sichuan, Chengdu, China; 2 School of Foreign Languages, Shanghai Ocean University, Shanghai, China; 3 School of Engineering and Technology, The Open University of Sichuan, Chengdu, China; 4 School of Education and Sport, Sichuan Vocational College of Health and Rehabilitation, Zigong, China; 5 School of Liberal Arts, Sichuan Winshare Vocational College, Chengdu, China

**Keywords:** AI-enhanced MOOCs, English proficiency, moderation analysis, technology acceptance model, vocational education

## Abstract

This study investigates whether English proficiency moderates core Technology Acceptance Model (TAM) pathways in the context of AI-enhanced English MOOCs for vocational students. Drawing on an extended TAM that links Perceived Ease of Use (PEOU), Perceived Usefulness (PU), Behavioral Intention (BI), and Perceived Learning Outcomes (PLO), we surveyed 516 learners from a provincial AI-powered MOOC. Confirmatory factor analysis confirmed strong measurement properties (all factor loadings > 0.74, AVE > 0.57, CR > 0.80). Structural analysis revealed robust direct effects: PEOU → PU (*β* = 0.756), PU → BI (*β* = 0.696), and BI → PLO (*β* = 0.814). Hierarchical regression showed no significant moderating by English proficiency on any TAM path, though a small positive direct effect on BI was observed (*β* = 0.064, *p* = 0.042). Results suggest that well-designed AI personalization can mitigate language-related barriers, allowing core TAM mechanisms to operate consistently across proficiency levels. The findings highlight the potential of adaptive AI tools to foster equitable engagement in vocational language learning. Future research should employ multi-item or objective proficiency measures and incorporate actual usage data to further validate these insights.

## 1 Introduction

The integration of Artificial Intelligence (AI) into educational technology has catalyzed a paradigm shift, particularly within the domain of language learning. AI-enhanced Massive Open Online Courses (MOOCs), equipped with adaptive algorithms, intelligent tutoring systems, and conversational agents, promise unprecedented levels of personalization, accessibility, and interactive engagement (Xia et al., 2024; Yu et al., 2017). This evolution holds profound significance for Vocational Education and Training (TVET), where upskilling in a globalized digital economy increasingly necessitates both technical competencies and English language proficiency (Ye et al., 2024). Consequently, understanding the drivers of successful adoption for these AI-enhanced platforms within vocational contexts is a pressing research imperative.

The Technology Acceptance Model (TAM) provides a robust theoretical lens for explaining users’ behavioral intentions toward technology, with Perceived Usefulness (PU) and Perceived Ease of Use (PEOU) consistently predicting adoption across diverse educational settings (Scherer et al., 2019; Xue et al., 2024). Extensions of TAM have fruitfully incorporated individual difference variables—such as learning motivation, self-regulation, and subjective norms—to capture nuanced adoption processes (Şimşek et al., 2025; Ye et al., 2023). Concurrently, empirical evidence robustly supports the efficacy of AI tools in language learning, demonstrating their capacity to reduce anxiety, enhance engagement, and improve proficiency through personalized, low-pressure practice environments (Yuan, 2024; Koç and Savaş, 2025).

Vocational learners operate at the intersection of occupation-specific technical training and the growing demand for English proficiency in transnational labor markets (Chen and Binti, 2026). When such learners engage with AI-enhanced MOOCs delivered in English, they confront a distinctive tension: the cognitive and affective load of processing technical content through a second language medium while exercising self-directed control over complex, often modularized instruction (Zheng et al., 2025). This dual demand amplifies the heterogeneity of their learning experiences, making it methodologically limiting to treat vocational cohorts as linguistically or motivationally uniform in technology acceptance research.

However, a critical gap persists. While research acknowledges the role of individual differences, studies have largely treated learner cohorts as homogeneous or focused on language proficiency as a learning outcome rather than as a potential antecedent shaping the acceptance process itself (Yuan, 2024; Tapalova and Zhiyenbayeva, 2022). This oversight is particularly salient in vocational AI-enhanced MOOCs, where learners with varied English abilities engage in self-directed, complex learning. Theoretically, English proficiency could act as a key boundary condition. From a cognitive load perspective, lower proficiency may increase the intrinsic load of navigating English-mediated interfaces, potentially attenuating PEOU and its translation into PU (Sweller, 2011; Zhang and Dong, 2024). From a motivational standpoint, perceived competence—a core need in Self-Determination Theory—is tied to language ability, which may influence how usefulness translates into behavioral intention (Ryan and Deci, 2000; Yuan and Liu, 2025).

Despite these plausible mechanisms, the explicit investigation of English proficiency as a moderator within the TAM framework for AI-enhanced vocational MOOCs remains underexplored. This study addresses this gap by posing a precise and critical question: Does English Proficiency Matter? Specifically, we investigate its role as a moderator within an extended TAM that links acceptance to Perceived Learning Outcomes (PLO). We hypothesize that English proficiency moderates the core TAM pathways (PEOU→PU, PU → BI, BI→PLO), with relationships being stronger for higher-proficiency learners.

By testing these hypotheses through a survey of vocational students using an AI-enhanced English MOOC, this study aims to make dual contributions.

(i) Theoretically, we position vocational learners as a boundary condition that extends TAM and offers an alternative account of AI-adaptivity as a mechanism that can buffer or substitute for the effects of English proficiency in shaping perceived usefulness, ease of use, and intention. By demonstrating that English proficiency systematically moderates key TAM pathways in AI-enhanced vocational MOOCs, we provide an alternative account of AI-adaptivity—one that incorporates linguistic competence and occupational constraints as critical contingencies shaping perceived usefulness, ease of use, and intention. This reframes AI-adaptivity not merely as algorithmic personalization, but as responsiveness to psychologically bounded learner profiles.
(ii) Practically, we generate design heuristics specifically for the “low-entry English proficiency + high occupational demand” niche, such as reducing initial linguistic barriers, scaffolding English input alongside domain content, and aligning interface feedback with time-constrained vocational schedules, to guide developers and educators in creating more equitable AI-MOOC environments.

## 2 Literature review

The convergence of AI and MOOCs offers transformative potential for Vocational Education and Training (TVET), where scalable, personalized upskilling—often requiring English proficiency—is vital (Ye et al., 2024). Whereas the Introduction has outlined the broader significance, this section focuses on synthesizing evidence linking AI personalization, technology acceptance, and learner heterogeneity to justify our moderation hypothesis.

### 2.1 AI-enhanced personalization and differential learner benefits

Research confirms AI tools (e.g., chatbots, intelligent tutors) enhance EFL learners’ oral proficiency, motivation, and engagement by reducing anxiety and enabling low-pressure interaction (Yuan, 2024; Koç and Savaş, 2025). Data-driven personalization improves learning efficiency and completion rates (Xia et al., 2024; Song et al., 2024), positioning learners as collaborative agents in human-AI interaction (Wang et al., 2024). These tools can also serve as cognitive “mind tools” that scaffold metacognitive planning and strategy use in self-regulated learning (Jin et al., 2023).

However, much of this literature assumes uniform benefit, overlooking that AI interfaces and content are often English-dominant. While advantages like individualized pacing and immediate feedback are well-documented (Schafer, 2025; Tapalova and Zhiyenbayeva, 2022), challenges persist, including risks to critical thinking development and occasional tool inaccuracies, which may affect learners differentially (Schafer, 2025). Empirical samples tend to focus on intermediate-to-advanced or higher education cohorts (Wang et al., 2024), and few tools are explicitly designed for low-proficiency learners (Koç and Savaş, 2025). Learner-centric studies reveal nuanced perceptions of AI integration, suggesting advantages are not equally accessible (Dai and Liu, 2024). Thus, pre-existing language competence may filter the perceived value and usability of AI tools, making proficiency a potential boundary condition for acceptance.

### 2.2 Technology acceptance and individual differences in TVET

TAM and UTAUT (Unified Theory of Acceptance and Use of Technology) remain dominant frameworks for predicting educational technology adoption. Meta-analyses confirm PU and PEOU as core predictors (Scherer et al., 2019; Xue et al., 2024), with extensions incorporating individual differences such as motivation and self-regulation (Şimşek et al., 2025). SDT (Self-Determination Theory) and S-O-R (Stimulus–Organism–Response) models further explain how autonomy and contextual support shape attitudes and outcomes (Liu et al., 2025; Yuan and Liu, 2025). Within vocational education, technology acceptance is recognized as a cornerstone for sustainable educational development (Ye et al., 2024). Unlike the Introduction, which introduced these theories briefly, we here emphasize their implication for vocational learners: expectancy-value beliefs and perceived relevance to practical skills uniquely influence acceptance (Ye et al., 2023), and prior AI experience shapes usage intentions (Hiniz, 2024). Crucially, English proficiency--essential for interacting with English-medium AI tools—has been understudied as a moderator of core TAM paths despite theoretical grounding in cognitive load and SDT.

### 2.3 Methodological foundations for moderation analysis

Testing moderation in SEM requires matching strategy to moderator type. For continuous moderators, latent interaction approaches capture non-additive effects while accounting for measurement error (Hair et al., 2021). For categorical moderators, multi-group analysis (MGA) is suitable, but its validity requires prior measurement invariance; these procedures are detailed in the Method section to keep the review focused on theory.

Control variables must be theoretically justified (Becker, 2005). In English-medium AI MOOCs, prior English proficiency and gender may affect perceived ease of use and usefulness via language-related cognitive load and gender differences in digital engagement (Sweller, 2011; Venkatesh and Davis, 2000), so they were included to isolate focal effects.

Adapting TAM scales to AI-mediated vocational contexts needs rigorous psychometric evaluation; specific EFA and invariance procedures are in the Method section, preserving the review’s conceptual focus.

This section outlines theoretical principles, not procedural steps, underpinning our moderation hypotheses.

### 2.4 Learner heterogeneity in MOOCs and proficiency as filter

MOOCs, especially in vocational education, encompass diverse designs—from structured xMOOCs to connectivist cMOOCs and hybrids balancing structure with autonomy (Anders, 2015). Their voluntary, self-directed nature amplifies learner heterogeneity, where motivation, self-regulation, and prior knowledge drive engagement and completion (Wei et al., 2024). AI personalization aims to address this diversity through adaptive paths and analytics (Yu et al., 2017; Song et al., 2024). Yet effectiveness varies with learner traits. Configurational research shows learning outcomes emerge from interactions among tool, task, and learner dimensions, with language ability pivotal (Zhang and Dong, 2024). In AI-enhanced vocational English MOOCs, English-dominated instruction may impose higher intrinsic load on low-proficiency learners (Sweller, 2011), attenuating PEOU→PU, and undermine perceived competence (Ryan and Deci, 2000), weakening PU → BI and BI→PLO. Thus, openness and equity promised by MOOCs may be moderated by proficiency.

### 2.5 Conclusion and focused research gap

Research recognizes AI’s personalization potential, TAM’s centrality in adoption studies, and the need for rigorous moderation in heterogeneous MOOC contexts. Perceived value and usability vary with learner traits. Though studies note differences in self-efficacy, digital literacy, or motivation, language proficiency is treated only peripherally (Wang et al., 2024; Koç and Savaş, 2025), with no work positioning English proficiency as a key TAM pathway moderator in AI-enhanced vocational MOOCs.

Vocational learners face a double jeopardy: structural barriers (time constraints, workplace demands, unequal digital access) and high MOOC-induced attrition, magnifying equity gaps. Without identifying moderators such as English proficiency, drivers of disengagement and dropout may remain unaddressed, making moderation both statistically necessary and pedagogically urgent.

Drawing on cognitive load and SDT perspectives, our study tests moderation effects to clarify whether proficiency alters acceptance mechanisms, advancing a nuanced, contingency-based view and informing equitable, proficiency-aware AI learning design in global vocational education.

## 3 Theoretical framework and hypotheses development

The study anchors in the Technology Acceptance Model (TAM) (Davis, 1989), positing that Behavioral Intention (BI) to use a system is driven by Perceived Usefulness (PU) and Perceived Ease of Use (PEOU). In education, the core path (PEOU → PU → BI) explains significant variance in e-learning acceptance (Scherer et al., 2019). Aligning with calls to link adoption to learning gains, we extend TAM by adding Perceived Learning Outcomes (PLO) as the final dependent variable (Venkatesh and Davis, 2000), positing that strong BI fosters higher PLO via sustained engagement (Wei et al., 2024). We thus propose:

*H1*: PEOU positively influences PU.

*H2*: PU positively influences BI.

*H3*: BI positively influences PLO.

The central contribution of this study is to examine English proficiency (EP) as a moderator of key TAM paths. Individual differences are known to alter TAM path strength (Venkatesh et al., 2003), and in AI-enhanced vocational English MOOCs, EP shapes learners’ cognitive-affective evaluations of the system (Zhang and Dong, 2024). Drawing on cognitive load theory (Sweller, 2011), we argue that lower EP increases intrinsic load when processing English interfaces and content, which can attenuate the PEOU→PU path; conversely, higher EP eases linguistic demands and strengthens this link (Song et al., 2024).

*H4*: English proficiency (EP) will significantly amplify or attenuate the strength of the path from perceived ease of use (PEOU) to perceived usefulness (PU). According to Self-Determination Theory (Ryan and Deci, 2000), higher EP enhances learners’ perceived competence and reduces cognitive load in processing English-medium content, thereby amplifying the positive impact of PEOU on PU. Conversely, lower EP may attenuate this path, as limited linguistic fluency can hinder recognition of system usefulness despite ease of navigation, leading to weaker PU formation (Yuan and Liu, 2025).

*H5*: English proficiency (EP) will significantly amplify or attenuate the path from behavioral intention (BI) to perceived learning outcomes (PLO). Higher EP may amplify the translation of intention into outcomes by enabling more effective comprehension and engagement with English-rich instructional materials, thus enhancing PLO (Scherer et al., 2019). Alternatively, lower EP may attenuate this relationship, as linguistic barriers can disrupt actual learning execution even when intention is high, resulting in diminished PLO (Venkatesh and Davis, 2000).

*H6*: English proficiency (EP) will significantly amplify or attenuate the path from perceived usefulness (PU) to behavioral intention (BI). Higher EP may amplify this path, as learners who are proficient in English are more likely to trust and commit to an AI-MOOC they perceive as useful, strengthening BI (Venkatesh and Davis, 2000). In contrast, lower EP may attenuate the path, since perceived usefulness might not readily translate into intention when learners anticipate excessive language-related effort, thereby weakening BI despite recognizing instrumental value (Scherer et al., 2019).

**Figure 1:**
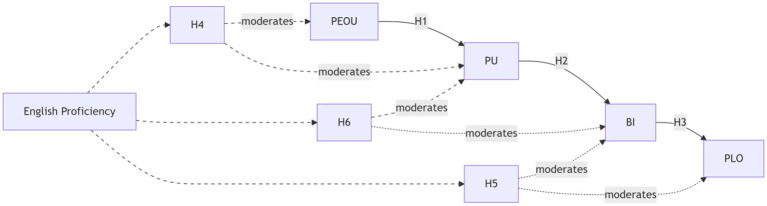
Hypothesized research model. Solid arrows represent hypothesized direct effects. Dashed arrows represent the hypothesized moderating effects of English proficiency (EP).

The research model is in Figure 1.

## 4 Methodology

### 4.1 Research design and data collection

This study adopted a quantitative, cross-sectional survey design to examine the moderating role of English proficiency (EP) within an extended Technology Acceptance Model (TAM) in AI-enhanced MOOCs for vocational education. This design enables testing of hypothesized causal and moderation effects in a naturalistic, large-scale setting (Hair et al., 2021; Scherer et al., 2019).

The target population comprised students, aged 19–20, primarily first-year and second-year students from a higher vocational college. These students were enrolled in a provincial-level AI-enhanced English as a Foreign Language (EFL) MOOC, College English, which integrates adaptive learning algorithms, intelligent tutoring, and personalized feedback (Xia et al., 2024; Yu et al., 2017). The course had completed ten iterations; the tenth enrolled *N* = 4,120 students from a three-year vocational college in China, with cumulative enrollment >17,000, reflecting its established relevance (Ye et al., 2024).

Data were collected online during the active learning phase of the tenth iteration via a structured, self-administered questionnaire hosted on WJX.cn. The survey link and participant information sheet (covering purpose, anonymity, voluntary participation) were distributed through the MOOC platform’s notification system. Electronic informed consent was obtained prior to questionnaire access. The survey remained open for two weeks. A total of 516 complete, valid responses were received (response rate ≈12.5%). This sample size exceeds the minimum recommended subject-to-item ratio for SEM and MGA (Costello and Osborne, 2005; Hair et al., 2021) and was pre-determined based on power analysis aligned with the number of items to ensure stable parameter estimation. To assess potential non-response bias, we compared early and late respondents on key variables; results indicated no significant differences (see Table 1 footnote), supporting the representativeness of the sample within the chosen vocational context.

**Table 1:** Exploratory factor analysis (EFA) results and item purification for each construct.

Construct	Initial items	Removed item & reason	Final items	KMO	Bartlett’s test (*p*)	Variance explained (%)	Factor loadings
Perceived Usefulness (PU)	4	*Item 3-R*: Low communality (0.284), negative loading (−0.533)	PU1, PU2, PU4	0.704	< 0.001	72.93	0.816–0.873
Perceived Ease of Use (PEOU)	4	*Item 6-R*: Low communality (0.275), negative loading (−0.524)	PEOU5, PEOU7, PEOU8	0.703	< 0.001	71.00	0.829–0.865
Behavioral Intention (BI)	4	*Item 11-R*: Very low communality (0.047), weak negative loading (−0.216)	BI9, BI10, BI12	0.716	< 0.001	73.17	0.843–0.865
Perceived Learning Outcomes (PLO)	5	*Item 14-R*: Low communality (0.182), negative loading (−0.426)	PLO13, PLO15, PLO16, PLO17	0.831	< 0.001	72.50	0.835–0.867

### 4.2 Measures and instrument development

All constructs were measured with multi-item reflective scales adapted from established instruments, using a 5-point Likert scale (1 = Strongly Disagree, 5 = Strongly Agree). Original English scales were translated into Chinese via back-translation by two bilingual researchers and reviewed by an educational technology expert for vocational student relevance.

*Perceived ease of use (PEOU) & perceived usefulness (PU)*: Adapted from Davis (1989), contextualized to “AI features in this MOOC” and “English learning.” Each initially had four items, including one reverse-coded item to reduce acquiescence bias.

*Behavioral intention (BI)*: Adapted from Venkatesh and Davis (2000), contextualized to continued use of the AI-MOOC for English learning, initially four items with one reverse-coded. As a methodological note, this study relies on behavioral intention rather than actual system usage data; although many MOOC platforms collect log data on user activities, such data were not available for this study, and future research could benefit from integrating such objective metrics to validate intention–behavior links.

*Perceived learning outcomes (PLO)*: Based on Wei et al. (2024) and Yuan and Liu (2025), five items captured self-reported knowledge acquisition, skill improvement, and learning effectiveness, including one reverse-coded item.

*English proficiency (EP)*: As the key moderator, given the constraints of a large-scale survey, a single self-report item (“How would you rate your overall English proficiency?”) was used (1 = Beginner, 5 = Advanced), justified for broad self-evaluative constructs in large surveys (Wanous et al., 1997; Postmes et al., 2013). While this approach facilitates straightforward data collection, it may not adequately capture the multidimensional nature of language competence and is susceptible to measurement error and restricted variance, which poses risks to construct validity and statistical conclusion validity—particularly in moderation analyses that are sensitive to such issues. Scores were mean-centered for interaction tests and categorized into Low (1–2), Intermediate (3), High (4–5) for group comparisons. The high-proficiency group was notably small (*n* = 18), substantially reducing statistical power and limiting the sensitivity of moderation tests. We note this as a potential limitation and recommend the use of multi-item, standardized proficiency measures in future research to enhance precision and statistical power.

Given the new context (AI-enhanced vocational MOOCs), we conducted psychometric validation on the main sample (*N* = 516). Exploratory Factor Analysis (EFA; Maximum Likelihood, Promax rotation) on the initial 17 items showed problematic cross-loadings/low communality (<0.40) for reverse-coded items, which is a common difficulty with reverse-coded items in self-report instruments (DiStefano and Motl, 2006). These four items were removed, leaving 13 positively worded items (PEOU:3; PU:3; BI:3; PLO:4). Final EFA yielded a clear four-factor structure with item loadings >0.70 and cross-loadings <0.40. Inter-item correlations (0.15–0.50) indicated good homogeneity without redundancy (Clark and Watson, 2019). Full item wording and sources appear in Appendix A.

### 4.3 Data analysis strategy

Analysis proceeded in three phases to ensure rigorous hypothesis testing:

*Phase 1 – measurement model validation*: Descriptive statistics and Cronbach’s alpha were computed for the multi-item constructs (PEOU, PU, BI, PLO); for the single-item English Proficiency (EP) measure, only descriptive statistics were reported, as internal consistency reliability is not applicable to single-item scales. Confirmatory Factor Analysis (CFA) in SPSSAU assessed model fit: χ^2^/df < 3, CFI > 0.90, TLI > 0.90, RMSEA <0.08 (Hair et al., 2021). Convergent validity was supported by factor loadings >0.70, AVE > 0.50, CR > 0.70; discriminant validity was checked via the Fornell-Larcker criterion. EP was treated as an observed variable in the structural model. It should be noted that prior AI experience and digital literacy were not measured in this study due to constraints on survey length and scope, and therefore could not be included as covariates or moderators in the analysis.

*Phase 2 – structural model & main effects (H1–H3)*: Path analysis within SEM tested direct paths PEOU → PU (H1), PU → BI (H2), BI → PLO (H3). EP was included as a control for potential direct effects. Path significance was assessed via bootstrapping (5,000 resamples).

*Phase 3 – moderation tests (H4–H6)*: EP was treated as a 3-level categorical variable (Low, Intermediate, High). Moderation analyses employed hierarchical regression with interaction terms. Given the use of an observed single-item measure for the moderator and the exploratory nature of this study, we prioritized a straightforward approach that facilitates interpretation for applied researchers and educators.

While latent interaction SEM is recommended in many TAM-based moderation studies because it accounts for measurement error in latent constructs, it requires multi-item indicators and more complex estimation procedures. Given the use of a single-item English proficiency (EP) measure in this study and the associated measurement constraints described above, such an approach was not feasible. Hierarchical regression with interaction terms was therefore adopted to test moderation effects, prioritizing a straightforward analytical strategy given the exploratory nature of the work.

First, hierarchical linear regressions in SPSSAU tested each hypothesized interaction separately. For each model, Step 1 entered main effects of predictor and EP (dummy-coded, Low as reference); Step 2 added the mean-centered predictor × EP interaction. Significant ΔR^2^ and interaction coefficients indicated moderation.

If interactions were significant, Multi-Group Analysis (MGA) in SEM probed the nature of effects: sample split into proficiency groups, measurement invariance tested, then path coefficients compared across groups. A significant Δχ^2^ between constrained and unconstrained models indicated omnibus moderation; pairwise comparisons identified specific patterns. This two-step approach ensured both statistical rigor and interpretative clarity.

Potential common method bias was assessed using Harman’s single-factor test; procedural remedies were implemented during data collection (see Section 5.2 for results and justification).

### 4.4 Ethical considerations

Ethical standards for human participants were upheld. The study protocol was approved by the Academic Committee of the Open University of Sichuan (Approval No. SOU202502); all participants (aged ≥18) provided written informed consent electronically after being informed of the study’s purpose, voluntary participation, and right to withdraw at any time.

The study was classified as minimal risk, involving an anonymous online survey on educational technology use, in accordance with institutional guidelines for low-risk social science research. Data were collected anonymously, with no personal identifiers recorded, and were securely stored on password-protected servers to ensure confidentiality and compliance with applicable data protection regulations.

## 5 Results

### 5.1 Measurement model validation

Prior to testing the structural hypotheses, the psychometric properties of all measurement scales were assessed by EFA for purification followed by CFA for validation (see Appendix B for full EFA details).

#### 5.1.1 Scale purification via exploratory factor analysis

An EFA using Maximum Likelihood extraction with Promax rotation was conducted separately for each construct to ensure unidimensionality and refine the scales (Costello and Osborne, 2005). The detailed process and results of the exploratory factor analysis (EFA) for scale purification are presented in Appendix B. Items were considered for removal if they exhibited low communality (< 0.40), cross-loadings, or factor loadings contrary to the theoretical direction. The purification process and results are summarized in Table 1.

As shown in Table 1, one item was removed from each of the Perceived Usefulness (PU), Perceived Ease of Use (PEOU), and Behavioral Intention (BI) scales due to poor psychometric properties (e.g., low communality, negative loadings). The Perceived Learning Outcomes (PLO) scale retained four items. The EFAs on the purified scales yielded excellent results, with all KMO values exceeding 0.70, significant Bartlett’s tests (*p* < 0.001), and high variance explained by a single factor (ranging from 71.00 to 73.17%). All retained items demonstrated high factor loadings (>0.81), confirming robust unidimensional structures for all four constructs.

#### 5.1.2 Confirmatory factor analysis of the final model

A CFA was performed on the final 13-item measurement model (PU: 3 items; PEOU: 3 items; BI: 3 items; PLO: 4 items). The model demonstrated a good fit to the data: χ^2^/df = 2.095, CFI = 0.96, TLI = 0.95, RMSEA = 0.074 (Hair et al., 2021). Detailed fit indices and validity metrics are presented in Table 2.

**Table 2:** Confirmatory factor analysis (CFA) results for the final measurement model.

Category	Index	Value	Criterion	Judgment
Model fit	χ^2^/df	2.095	< 3.0	Good
	CFI	0.96	> 0.90	Good
	TLI	0.95	> 0.90	Good
	RMSEA	0.074	< 0.08	Good
Convergent validity	Standardized factor loadings	0.74–0.81	> 0.70	Good
	Average variance extracted (AVE)	PU: 0.60 PEOU: 0.57 BI: 0.60 PLO: 0.63	> 0.50	Good
	Composite reliability (CR)	All > 0.80	> 0.70	Good
Discriminant validity	Fornell-Larcker criterion	√ (Met)	√ AVE > Correlations	Established
Internal consistency reliability	Cronbach’s *α*	All > 0.90	> 0.70	Excellent

Final model comprised 13 items (PU = 3, PEOU = 3, BI = 3, PLO = 4). All factor loadings significant at *p* < 0.001. √ denotes √AVE > inter-construct correlations.

Non-response bias was assessed by comparing early (*n* = 129) and late (*n* = 129) respondents on key variables (Armstrong and Overton, 1977). Independent samples *t*-tests showed no significant differences for English proficiency (*p* = 0.761), PU (*p* = 0.172), BI (*p* = 0.070), or PLO (*p* = 0.071); a small-to-medium difference emerged for PEOU (*p* = 0.014, Cohen’s d = −0.32). These results suggest non-response bias is unlikely to seriously threaten sample representativeness.

Convergent validity, discriminant validity, and internal consistency reliability were all established (see Table 2). Specifically, standardized factor loadings exceeded 0.70, AVE > 0.50, CR > 0.80, √AVE > inter-construct correlations (Fornell and Larcker, 1981), and Cronbach’s *α* > 0.90 (Nunnally and Bernstein, 1994). The purified measurement model thus demonstrated strong psychometric properties, supporting subsequent hypothesis testing (Hair et al., 2021).

### 5.2 Descriptive statistics, correlations, and common method bias assessment

The descriptive statistics and inter-construct correlations for the final latent variables are summarized in Table 3. All constructs demonstrated mean scores above the theoretical midpoint of the scale (*M* = 3.64 to 3.74), suggesting that participants, on average, held positive perceptions regarding the AI-enhanced MOOC system across all dimensions measured.

**Table 3:** Descriptive statistics and correlations among key variables (dimension means).

Variable	*M*	SD	1	2	3	4
1. PEOU	3.736	0.655	1			
2. PU	3.689	0.678	0.763**	1		
3. BI	3.641	0.724	0.749**	0.718**	1	
4. PLO	3.663	0.713	0.691**	0.712**	0.816**	1

PEOU, Perceived Ease of Use; PU, Perceived Usefulness; BI, Behavioral Intention; PLO, Perceived Learning Outcomes. All variables represent the mean scores of their respective items (dimension means), *N* = 516. Pearson correlations are reported; **p < 0.01.

More critically, as presented in Table 3, the correlations among the four core constructs were all positive and statistically significant (*p* < 0.01). The strongest relationships were observed between Behavioral Intention (BI) and Perceived Learning Outcomes (PLO) (*r* = 0.816) and between Perceived Ease of Use (PEOU) and Perceived Usefulness (PU) (*r* = 0.763). These findings highlight strong interconnections among the constructs and provide preliminary, bivariate support for the extended TAM framework.

To assess potential common method bias, we employed procedural remedies during data collection (e.g., anonymous responses, temporal and psychological separation of items) and conducted Harman’s single-factor test. An unrotated principal component analysis of all 13 measurement items showed that the first factor explained 58.47% of the variance—above the conventional 50% threshold but below the 70% criterion proposed by Podsakoff et al. (2003).

Harman’s test has limited diagnostic accuracy with multiple correlated constructs and may overstate method bias (Spector, 2006). Our procedural safeguards align with established practices for reducing common method bias in self-report surveys (Podsakoff et al., 2003). Given that the variance remained below 70%, alongside these safeguards and the absence of unusually high inter-item correlations, the result suggests that common method bias is unlikely to pose a serious threat to the validity of the study (Podsakoff et al., 2003; Spector, 2006). Unrotated factor loadings are provided in Appendix C2 for transparency.

To preliminarily examine the differences across groups, the means and standard deviations of the key variables for each English proficiency level are presented in Table 4.

**Table 4:** Means and standard deviations of key variables by English proficiency level (dimension means).

Variable	Low (*n* = 344)	Intermediate (*n* = 154)	High (*n* = 18)	Total (*N* = 516)
	M/SD	M/SD	M/SD	M/SD
PEOU	3.720/0.675	3.736/0.591	4.037/0.749	3.736/0.655
PU	3.674/0.708	3.703/0.595	3.852/0.777	3.689/0.678
BI	3.609/0.738	3.682/0.678	3.926/0.788	3.641/0.724
PLO	3.628/0.727	3.719/0.662	3.847/0.845	3.663/0.713

See Table 3 for variable definitions. English proficiency groups were coded as: 1.0 = Low (*n* = 344), 2.0 = Intermediate (*n* = 154), 3.0 = High (*n* = 18). *N* = 516.

### 5.3 Hypothesis testing for direct effects (H1, H2, H3)

The hypothesized direct paths within the extended TAM framework were tested using hierarchical regression analysis, with English proficiency included as a control variable in all models (Table 5).

**Table 5:** Results of hierarchical regression analysis for testing direct effects (H1, H2, H3).

Hypothesis	DV	Predictor(s)	*β*	*t*	*p*	95% CI for *β*	R^2^	*F*	Supported?
H1 (PEOU → PU)	PU	*PEOU* English Proficiency	**0.756** −0.007	**26.023** −0.244	**< 0.001** 0.808	**[0.699, 0.813]** [−0.076, 0.059]	0.570	*F*(2, 513) = 340.534, *p* < 0.001	Yes
H2 (PU → BI)	BI	*PU* English Proficiency	**0.696** 0.064	**22.113** 2.041	**< 0.001** 0.042*	**[0.634, 0.758]** [0.003, 0.149]	0.494	*F*(2, 513) = 249.962, *p* < 0.001	Yes
H3 (BI → PLO)	PLO	*BI* English Proficiency	**0.814** 0.014	**31.743** 0.538	**< 0.001** 0.591	**[0.764, 0.865]** [−0.043, 0.076]	0.666	*F*(2, 513) = 511.249, *p* < 0.001	Yes

See Table 3 for variable definitions. *N* = 516 for all analyses. DV, dependent variable. *β* values are standardized regression coefficients. CI, confidence interval. English Proficiency was included as a control variable in all models. All VIF values were below 2, indicating no multicollinearity concerns. *p* values are two-tailed. Values in bold indicate statistical significance at the *p* <.001 level. This means the results are highly unlikely to occur by chance, providing strong evidence for the observed effects.

As shown in Table 5, all three direct effect hypotheses were supported.

*H1* (PEOU → PU) showed a strong positive effect (*β* = 0.756, *p* < 0.001, R^2^ = 0.570); English proficiency was non-significant (*β* = −0.007, *p* = 0.808).

*H2* (PU → BI) was also supported (*β* = 0.696, *p* < 0.001, R^2^ = 0.494), with a small but significant positive effect of English proficiency on BI (*β* = 0.064, *p* = 0.042).

*H3* (BI → PLO) received the strongest support (*β* = 0.814, *p* < 0.001, R^2^ = 0.666), while English proficiency had no significant direct effect on PLO (*β* = 0.014, *p* = 0.591).

In summary, robust empirical support was found for H1–H3, confirming the core TAM relationships—PEOU positively predicts PU, PU predicts BI, and BI predicts PLO—in the context of AI-enhanced MOOCs for vocational students.

### 5.4 Testing the moderating role of English proficiency (H4, H5, H6)

To examine whether English proficiency moderates the core relationships within the extended TAM (H4, H5, H6), hierarchical regression analyses with interaction terms were conducted. The continuous English proficiency variable was mean-centered prior to creating interaction terms to mitigate multicollinearity. The results of these analyses are summarized in Table 6.

**Table 6:** Results of moderation analysis for H4, H5, and H6.

Hypothesis	DV	Step	Predictor(s)	*β*	*t*	*p*	95% CI for *β*	ΔR^2^	f^2^	Observed power	Interpretation
H4 (Moderation: PEOU → PU)	PU	Step 1	PEOU English Proficiency	0.749** −0.004	25.438 −0.142	< 0.001 0.887	[0.692, 0.807] [−0.072, 0.063]	—	—	—	—
		Step 2	PEOU English Proficiency **PEOU × English**	0.749** −0.004 **−0.037**	25.438 −0.142 **−1.261**	< 0.001 0.887 **0.208**	[0.692, 0.807] [−0.072, 0.063] **[−0.103, 0.022]**	**0.001** (*p* = 0.208)	0.0047	0.28	Not significant; test under-powered (f^2^ < 0.005)
H5 (Moderation: PU → BI)	BI	Step 1	PU English Proficiency	0.695** 0.064*	21.759 2.041	< 0.001 0.042	[0.633, 0.758] [0.003, 0.149]	—	—	—	—
		Step 2	PU English Proficiency **PU × English**	0.695** 0.064* −0.003	21.759 2.041 **−0.092**	< 0.001 0.042 **0.927**	[0.633, 0.758] [0.003, 0.149] **[−0.070, 0.064]**	**0.000** (*p* = 0.927)	0.0040	0.05	Not significant; test under-powered (f^2^ < 0.005)
H6 (Moderation: BI → PLO)	PLO	Step 1	BI English Proficiency	0.810** 0.016	31.381 0.616	< 0.001 0.538	[0.759, 0.860] [−0.041, 0.078]	—	—	—	—
		Step 2	BI English Proficiency **BI × English**	0.810** 0.016 **−0.042**	31.381 0.616 **−1.630**	< 0.001 0.538 **0.104**	[0.759, 0.860] [−0.041, 0.078] **[−0.102, 0.010]**	**0.002** (*p* = 0.104)	0.0060	0.38	Not significant; low power (effect small)

See Table 3 for variable definitions. English Proficiency was mean-centered. ΔR^2^ derived from comparison of Step 2 and Step 1 models; f^2^ calculated as ΔR^2^/(1 − R^2^Step 2). Observed power computed using F test of the interaction term (df_1_ = 1, df_2_ = 512, *α* = 0.05).The three bolded rows correspond to Step 2 models that included interaction terms between English Proficiency (mean centered) and other predictors. These interaction terms were not statistically significant (*p* >.05), indicating that English Proficiency did not exert a significant moderating effect on the examined relationships.

A key consideration in interpreting these results is that the high-proficiency group was very small (*n* = 18), and all interaction effects were small (f^2^ ≤ 0.006; H4/H5 tests under-powered with f^2^ < 0.005). Consequently, the non-significant interactions should be interpreted as an absence of evidence for moderation, rather than evidence of no moderation.

**Figure 2:**
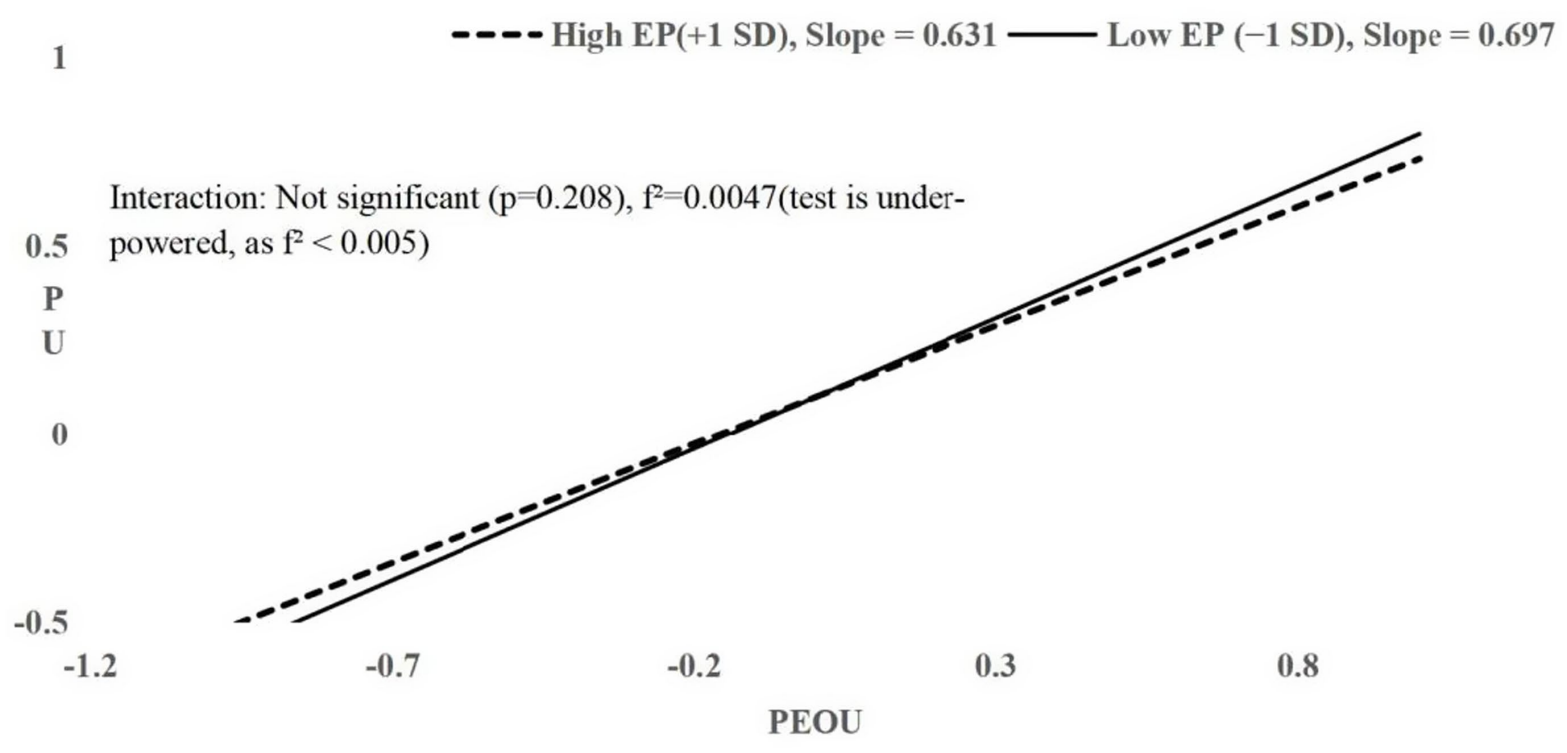
Simple slopes of PEOU on PU at high (+1 SD) and low (−1 SD) English proficiency. Near-parallel slopes indicate minimal moderation of the PEOU–PU relationship by EP.

**Figure 3:**
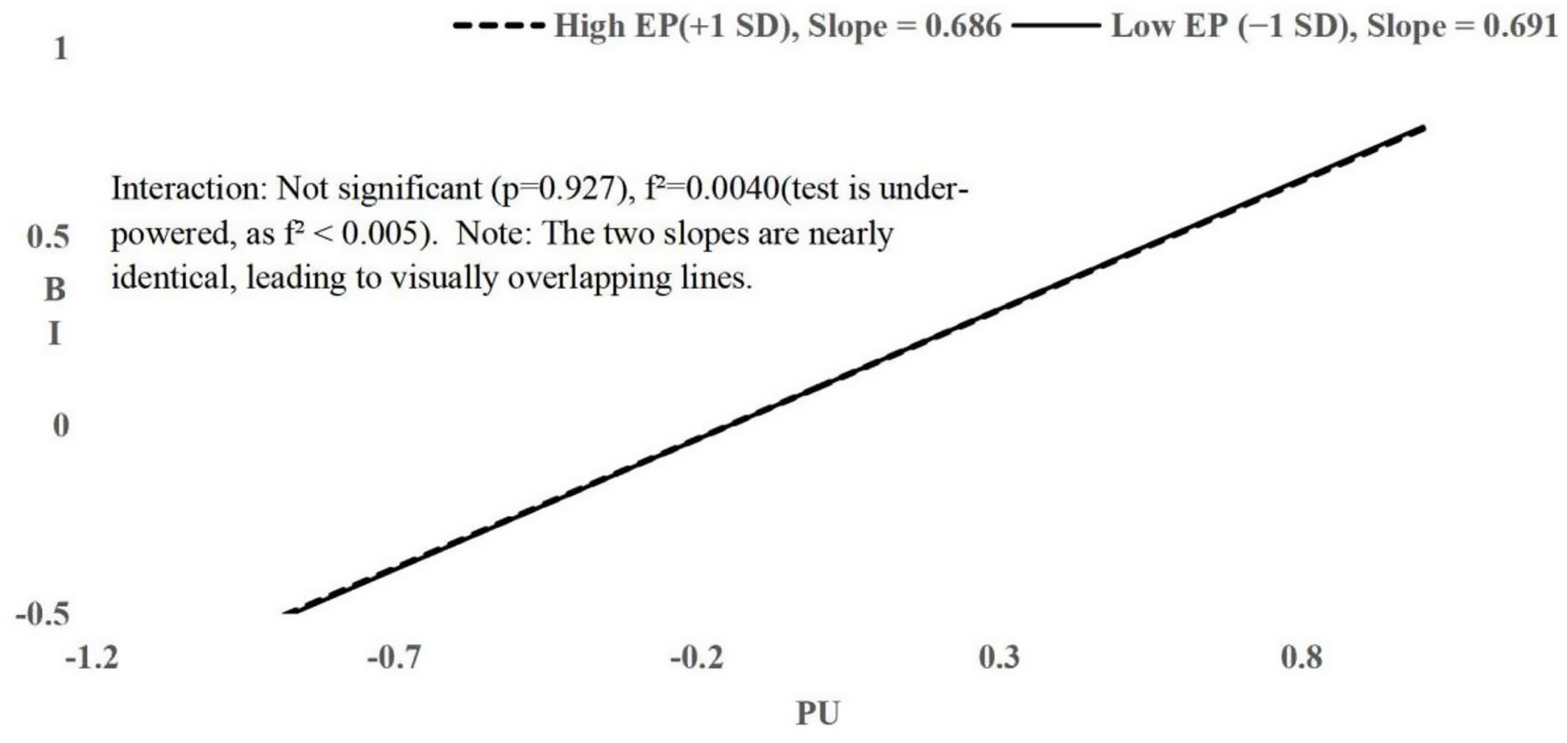
Simple slopes of PU on BI moderated by EP. Simple-slopes plot shows near-parallel lines at high (+1 SD: EP = 2.95, slope = 0.686) and low (−1 SD: EP = 1.31, slope = 0.691) proficiency, indicating only a negligible difference in the PU–BI relationship.

**Figure 4:**
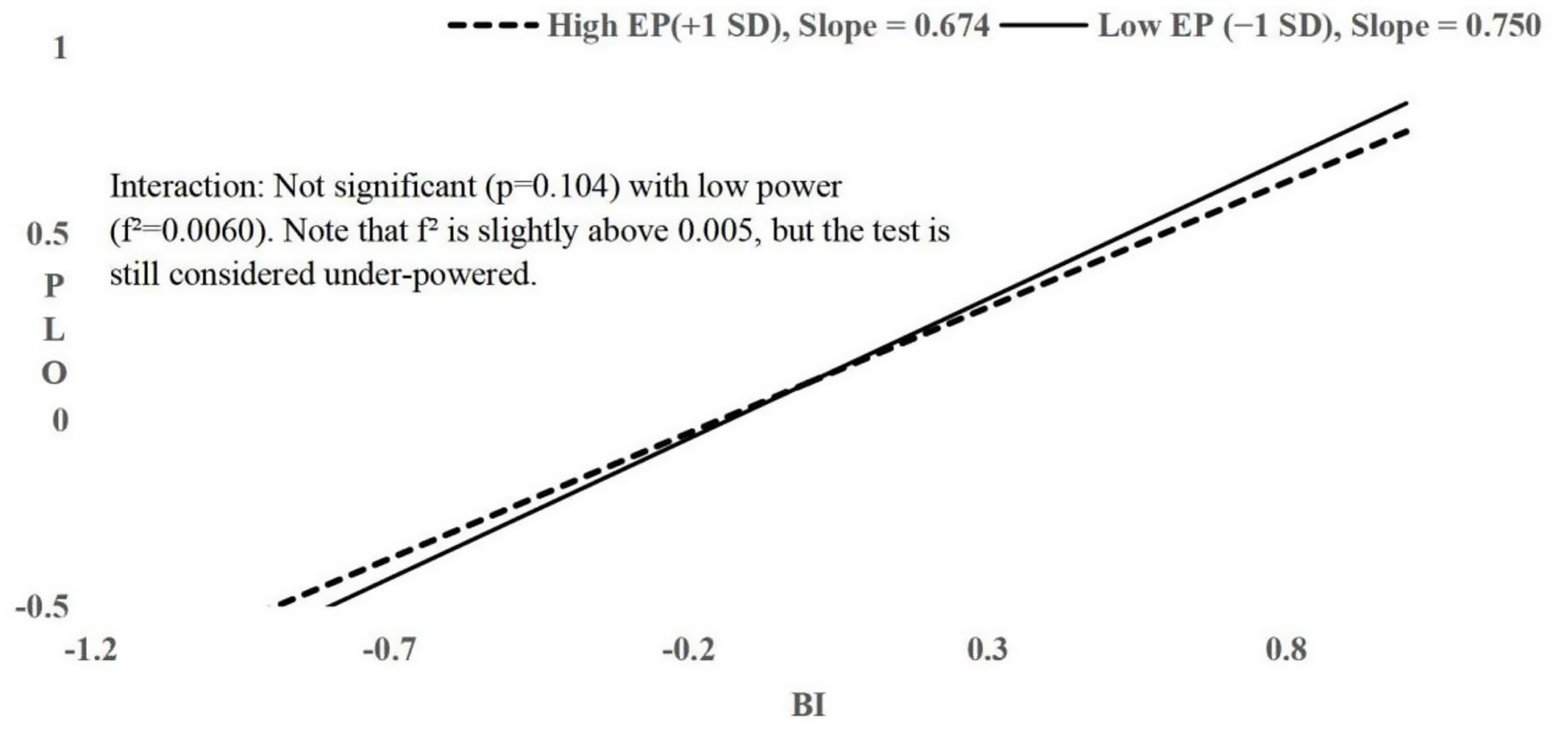
Simple slopes of PU on BI at high (+1 SD) and low (−1 SD) English proficiency. Near-parallel slopes indicate negligible moderation of the PU–BI relationship by EP.

All interactions had f^2^ ≤ 0.006; H4 and H5 had f^2^ < 0.005, indicating tests were under-powered to detect effects of this magnitude. Non-significance should not be interpreted as evidence of zero effect. Simple slopes plots (Figures 2–4) visualize conditional relationships at English Proficiency ±1 SD.

As shown in Table 6, the interaction terms for all three hypothesized moderation effects were statistically not significant.

#### 5.4.1 H4 (PEOU → PU moderation)

H4 proposed that English proficiency (EP) moderates the relationship between PEOU and PU. Hierarchical regression showed a strong main effect of PEOU on PU (*β* = 0.749, *t* = 25.438, *p* < 0.001, 95% CI [0.692, 0.807]) and a non-significant main effect of EP (*β* = −0.004, *t* = −0.142, *p* = 0.887, 95% CI [−0.072, 0.063]). Adding the interaction term (PEOU × EP) in Step 2 yielded *β* = −0.037, *p* = 0.208, ΔR^2^ = 0.001, f^2^ = 0.0047, observed power = 0.28, 95% CI for *β* = [−0.103, 0.022]. The 95% CI values for PEOU and EP in Step 2 remain the same as in Step 1 ([0.692, 0.807] for PEOU and [−0.072, 0.063] for EP), indicating no change in their main effects. Simple slopes at ±1 SD EP were near-parallel (high EP slope = 0.631, low EP slope = 0.697; Figure 2; calculation details in Appendix D), indicating minimal difference. H4 was not supported, though a small moderation effect cannot be ruled out given limited power and measurement precision.

#### 5.4.2 H5 (PU → BI moderation)

H5 hypothesized that English proficiency (EP) moderates the relationship between PU and BI. Hierarchical regression showed significant main effects of PU on BI (*β* = 0.695, *t* = 21.759, *p* < 0.001, 95% CI [0.633, 0.758]) and of EP on BI (*β* = 0.064, *t* = 2.041, *p* = 0.042, 95% CI [0.003, 0.149]). Adding the interaction term (PU × EP) in Step 2 yielded *β* = −0.003, *t* = −0.092, *p* = 0.927, ΔR^2^ ≈ 0.000, f^2^ = 0.0040, observed power = 0.05, 95% CI for *β* = [−0.070, 0.064]. Simple slopes at ±1 SD EP were near-parallel (high EP slope = 0.686, low EP slope = 0.691; Figure 3), showing only a negligible difference. H5 was not supported, but the effect may be too small to detect with the current sample and measurement precision.

#### 5.4.3 H6 (BI → PLO moderation)

H6 proposed that English proficiency (EP) moderates the relationship between BI and PLO. Hierarchical regression showed a strong main effect of BI on PLO (*β* = 0.810, *t* = 31.381, *p* < 0.001, 95% CI [0.759, 0.860]) and a non-significant main effect of EP (*β* = 0.016, *t* = 0.616, *p* = 0.538, 95% CI [−0.041, 0.078]). Adding the interaction term (BI × EP) in Step 2 yielded *β* = −0.042, *t* = −1.630, *p* = 0.104, ΔR^2^ = 0.002, f^2^ = 0.0060, observed power = 0.38, 95% CI for *β* = [−0.102, 0.010]. The 95% CI values for BI and EP in Step 2 remain the same as in Step 1 ([0.759, 0.860] for BI and [−0.041, 0.078] for EP), indicating no change in their main effects. Simple slopes at ±1 SD EP were near-parallel (high EP slope = 0.750, low EP slope = 0.674; Figure 4; calculation details in Appendix D), reflecting only a trivial difference across EP levels. H6 was not supported, but a modest moderating effect cannot be excluded given the small effect size, low power, and measurement limitations of the moderator.

#### 5.4.4 Conclusion on moderation analysis

The hierarchical regression analyses revealed no statistically significant moderating effects of English proficiency (EP) on the three hypothesized paths. Given the under-powered tests for some interactions (observed power ranging from 0.05 to 0.38), the use of a single-item moderator with limited variance, and the pronounced group imbalance—particularly the very small high-proficiency group (*n* = 18)—these results do not preclude the presence of small moderation effects. Consequently, the prerequisite for a meaningful multi-group analysis (MGA) was not met (Hair et al., 2021), and MGA was not conducted. The direct effects of PEOU on PU, PU on BI, and BI on PLO remained similar across English proficiency levels in this sample, as indicated by near-parallel simple slopes and minimal differences in effect sizes (see Figures 2–4), though the possibility of minor variation cannot be ruled out. We stress that the absence of significant moderation reflects limitations in measurement precision and statistical power, not definitive evidence of no moderation effect.

### 5.5 Direct effects of the control variable & post-hoc examination of group mean difference

In addition to testing its moderating role, the direct effects of English proficiency (the control variable) on the endogenous variables were examined. As shown in the regression results for direct effects (Table 5), its direct effect on Perceived Usefulness (PU: *β* = −0.005, *p* = 0.887) and Perceived Learning Outcomes (PLO: *β* = 0.019, *p* = 0.538) was non-significant. However, a small but statistically significant positive direct effect on Behavioral Intention (BI: *β* = 0.076, *p* = 0.042) was observed. This indicates that, while English proficiency did not moderate the TAM relationships as hypothesized, it had a modest independent association with students’ intention to use the AI-enhanced MOOC, though not with their perceptions of its usefulness or their reported learning outcomes.

Furthermore, to contextualize these findings, we present the descriptive statistics of the key constructs segmented by proficiency groups (Table 4). While mean scores for PEOU, PU, BI, and PLO generally showed a positive trend across proficiency levels from Low to High, the regression analyses reported earlier indicated that these group differences did not translate into statistically significant moderating effects on the core TAM pathways.

Post-hoc examination of group differences: Although the moderating effects of English proficiency were not statistically significant (as reported in Table 6), the descriptive statistics in Table 4 reveal notable mean-level differences across proficiency groups. Students with high English proficiency consistently reported the highest mean scores on all four constructs (PEOU, PU, BI, and PLO), followed by the intermediate and low proficiency groups. This pattern suggests that while the strength of the relationships among TAM constructs is invariant across groups, students with higher English proficiency may hold a more positive baseline perception of the AI-enhanced MOOC system. This finding warrants further investigation in future research, perhaps exploring other individual difference variables that might explain this baseline variation.

## 6 Discussion

This study examined whether English proficiency (EP) moderates core Technology Acceptance Model (TAM) pathways in an AI-enhanced English MOOC for vocational students. Contrary to hypotheses, EP did not significantly moderate the PEOU→PU, PU → BI, or BI→PLO paths (H4–H6). However, the foundational TAM relationships (H1–H3) were robustly supported, and a small but significant direct effect of EP on BI emerged.

### 6.1 Interpretation of key findings

The absence of moderation suggests that, within this AI-enhanced vocational MOOC, the psychological mechanisms linking technology perceptions to adoption and perceived learning operate consistently across proficiency levels. The robustness of PEOU→PU, PU → BI, and BI→PLO indicates that learners at all levels perceived the system’s ease of use, usefulness, and value similarly.

Two factors may explain the null moderation. First, the AI system’s adaptive algorithms and personalized feedback likely served as an equalizer, reducing intrinsic cognitive load for lower-proficiency learners when navigating English-mediated interfaces (Sweller, 2011; Xia et al., 2024; Song et al., 2024). This aligns with evidence that well-designed AI tools foster low-pressure, supportive environments benefiting diverse users (Yuan, 2024). One possible post-hoc account is that the AI system’s adaptive features help mitigate language-related cognitive load, thereby reducing proficiency differences in how learners engage with the MOOC. This explanation is theoretically reasonable; however, because our study did not assess participants’ perceived system adaptivity or related mechanisms, the claim remains speculative. Future work must therefore measure perceived adaptivity to examine this pathway directly.

Second, the vocational context’s strong goal-orientation—linking skill acquisition to career competency—may generate uniformly high perceived usefulness for a tool seen as instrumental to job-relevant English skills (Ye et al., 2024), overriding individual proficiency differences.

Support for the core TAM paths reaffirms the model’s validity in AI-powered vocational language MOOCs (Xue et al., 2024). The notably strong BI→PLO path (*β* = 0.814) highlights the pivotal link between acceptance and self-reported learning gains, supporting the extension of TAM to include learning outcomes (Wei et al., 2024; Yuan and Liu, 2025).

The small positive direct effect of EP on BI implies that higher-proficiency learners may begin with slightly stronger engagement intention, possibly due to greater initial confidence or lower apprehension in an English-dominant platform (Yuan, 2024). Yet this effect did not alter the strength of primary TAM relationships; once engaged, acceptance processes appeared similarly driven by perceived ease and usefulness for all.

### 6.2 Theoretical implications

This study challenges the presumed centrality of language proficiency as a key moderator in technology-mediated language learning under AI support. Although cognitive load theory (Sweller, 2011) and self-determination theory (Ryan and Deci, 2000) predicted moderation, results suggest that AI-driven systems in this context may buffer disadvantages of lower proficiency; as elaborated in Section 6.1, this may reflect the homogenizing influence of adaptive capabilities such as personalized content and scaffolded feedback. However, this null finding should not be interpreted as definitive evidence of no moderation, nor as confirmation that personalization eliminates proficiency effects, since such mechanisms were not directly measured in the present study.

Two perspectives may help explain this null finding. First, a “main-effect” view posits that the system’s adaptive capabilities could minimize conditional impacts of pre-existing traits like language skill, leading to uniform acceptance across users. Recent AI–education fit research provides direct empirical anchor for this perspective: Xia et al. (2024) demonstrated across Edmodo and Duolingo that real-time adaptive content significantly reduced mastery time and increased retention irrespective of learner background; Qu (2025) found proficiency levels served as a weak mediator of learning outcomes in AI-driven adaptive EFL platforms; and Hong and Guo (2025) provided an empirical study demonstrating consistent benefits of AI-enhanced systems across diverse learner profiles in applied educational settings, providing direct evidence for the person–technology fit perspective. Together, these studies indicate that algorithmic personalization can attenuate proficiency-related disparities, aligning with our observation of consistent TAM pathways across English levels.

Second, person-technology fit and adaptive structuration theories suggest that high perceived adaptivity allows the AI systems to accommodate diverse proficiencies, or that learners across levels appropriate features comparably to meet goals (Goodhue and Thompson, 1995; DeSanctis and Poole, 1994). This view is reinforced by Zhang and Dong (2024), who integrated TAM, cognitive load theory, and HCI (human–computer interaction) in a hybrid fsQCA–system dynamics study of GenAI in English learning. Their analysis revealed complex, non-linear interactions among language ability, motivation, and ethical concerns, underscoring that acceptance pathways can remain relatively uniform when AI systems provide sufficient adaptivity.

Furthermore, the vocational setting’s pragmatic orientation appears to foster acceptance robust to individual language differences. Hong and Guo (2025) experimentally demonstrated that AI-enhanced multi-display language teaching systems significantly improved motivation, cognitive load management, and learner autonomy among EFL students regardless of baseline differences, reinforcing the idea that well-designed AI environments can yield consistent benefits across learner profiles in applied educational settings. This resonates with research on vocational learners’ unique motivational drivers (Ye et al., 2023), indicating that perceived instrumental value for career-relevant competencies may outweigh individual traits in driving acceptance.

Thus, while “system adaptability” or “perceived personalization” emerge from the literature as promising constructs that may help explain uniform acceptance patterns, they were not directly examined in this study. We therefore position them as a promising direction for future research to clarify when and how technology design mitigates stable learner traits and promotes universal acceptance patterns. Integrating perceived adaptivity into extended TAM or UTAUT frameworks (Yu et al., 2017) offers a viable avenue for such inquiry.

### 6.3 Practical implications

For designers, developers, and vocational educators, the findings imply that well-implemented AI-enhanced features—adaptive pathways, intelligent feedback, personalized content—can foster inclusive environments where core TAM pathways function similarly across English levels. Communicating ease of use and career utility positively influences all students’ intention and learning perceptions, regardless of initial proficiency.

However, the small direct effect of EP on BI and descriptively higher intention among high-proficiency learners signal that initial entry barriers may differ. Support should therefore target lowering initial thresholds and shaping positive first impressions, rather than modifying the stable acceptance mechanism.

Proficiency-sensitive onboarding strategies are recommended: e.g., platform orientation in learners’ first language, increased visual/multimodal support in the AI interface, or supplementary foundational language resources alongside the MOOC (Koç and Savaş, 2025). These steps aim to ensure all learners initially perceive the system as useful and easy to use, entering the positive acceptance cycle; thereafter, AI adaptivity and robust TAM pathways sustain engagement and foster perceived learning.

Beyond pedagogical efficacy, the model also offers economic advantages in scaled deployment: such AI-MOOCs exhibit declining marginal costs yet increasing language-support returns at scale, yielding superior cost-effectiveness in vocational upskilling. As learner numbers grow, the fixed development costs of adaptive modules are distributed across more users, lowering per-learner expense, while personalized language support continues to improve outcomes, making the approach particularly advantageous for large-scale vocational training initiatives.

### 6.4 Limitations and future research

Several limitations should be noted.

(1) Measurement limitations: English proficiency (EP) was assessed with a single-item, 5-point self-report, limiting the capture of multifaceted proficiency and increasing measurement error, which weakened tests of moderation (H4–H6); the absence of significant effects should thus be interpreted as an absence of evidence rather than evidence of no effect. During psychometric validation, all reverse-coded items were removed to resolve cross-loadings, leaving only positively worded items, which may introduce acquiescence bias. Moreover, AI-adaptivity—conceptualized as the system’s capacity to buffer linguistic demands (e.g., adaptive scaffolding, real-time feedback, simplified input)—was not directly measured because data collection preceded the development of multi-dimensional indicators. Consequently, we could not empirically adjudicate the core claim that AI-adaptivity may substitute for EP in mitigating language-related cognitive load. Future studies should employ multi-item or objective EP measures and develop validated, multi-dimensional indicators for AI-adaptivity to enable a direct test of this substitution hypothesis.

(2) Analytical and sample constraints: The moderation analysis relied on hierarchical regression with a single-item moderator and was further constrained by a pronounced imbalance across proficiency groups, particularly the very small high-proficiency group (*n* = 18), reducing statistical power and sensitivity to detect moderation. In addition, the sample was drawn from a single Chinese vocational college and a specific AI-enhanced MOOC platform, explicitly limiting cross-cultural and cross-platform generalizability. While the findings offer insights into this context, caution should be exercised in extending them to other cultural settings or AI-based learning systems. Comparative studies across cultures, educational levels, and AI tool types are needed to assess broader applicability.

(3) Unmeasured individual differences: Due to constraints on survey length and scope, other potentially relevant individual differences—such as digital literacy, learning agility, prior AI experience, and motivational orientations (Şimşek et al., 2025; Liu et al., 2025)—were not measured and therefore could not be examined as covariates or moderators. These factors may moderate TAM pathways and merit exploration in future research.

(4) Cross-sectional design: The use of cross-sectional data precludes causal inference about the directional relationships among TAM constructs. Although longitudinal research tracking proficiency growth, acceptance perceptions, and actual usage over time is needed, future studies should specifically adopt a cross-lagged panel design combined with objective learning analytics (e.g., system log data on engagement and performance) to examine bidirectional effects and link acceptance with verifiable learning behaviors.

In conclusion, core TAM tenets held strongly in this AI-enhanced vocational English MOOC context. While EP showed a minor direct link to intention, it did not moderate key acceptance pathways. Given measurement limitations and reduced statistical power from group imbalance, this null moderation finding is preliminary and represents an absence of evidence rather than definitive proof of no effect. The findings suggest that well-designed adaptive AI tools may transcend initial language barriers, fostering more equitable technology acceptance in vocational education. The democratizing potential of AI warrants continued investigation into its interplay with individual differences and learning contexts.

## 7 Conclusion

This study set out to investigate a critical question for inclusive educational technology: whether English language proficiency acts as a significant boundary condition in the acceptance of AI-enhanced MOOCs among vocational students. Grounded in an extended Technology Acceptance Model (TAM), the findings offer a nuanced and ultimately optimistic perspective, though they must be contextualized within the study’s methodological boundaries.

The results robustly confirm the enduring explanatory power of the core TAM relationships in this novel context. Perceived Ease of Use strongly predicted Perceived Usefulness, which in turn was a key driver of Behavioral Intention, and this intention was a potent antecedent of Perceived Learning Outcomes (Davis, 1989; Scherer et al., 2019). Crucially, however, our central hypothesis was not supported: English proficiency did not significantly moderate any of these key pathways (PEOU→PU, PU → BI, BI→PLO). It is essential to note that this key test of moderation relied on a single-item self-report measure of proficiency, which limits the depth and reliability of this specific finding. Nevertheless, the pattern suggests that the psychological mechanisms driving technology acceptance for this AI-enhanced tool may operate with considerable consistency across learners with varying language abilities (Şimşek et al., 2025; Ye et al., 2023).

The primary theoretical implication is that the “boundary condition” of language proficiency may be more malleable than previously theorized when advanced, well-designed educational technology is involved (Zhang and Dong, 2024). While cognitive load and self-determination theories provide a rationale for expecting moderation (Sweller, 2011; Ryan and Deci, 2000), the AI-powered personalization and scaffolding inherent in the MOOC may have effectively mitigated language-related barriers, equalizing perceptions of utility and ease of use (Xia et al., 2024). This study proposes that in highly intelligent learning environments, technology design features could potentially redefine the impact of individual differences, and future research should investigate incorporating constructs like “system adaptability” into theoretical models.

Practically, this is an encouraging finding for instructional designers and educators. It indicates that investing in intuitive, useful AI features can create equitable learning environments where core acceptance drivers function effectively for a linguistically diverse population. The tool’s design and perceived vocational relevance appear paramount. Nonetheless, the observed small direct effect of proficiency on Behavioral Intention suggests that supplementary, proficiency-sensitive onboarding or support could further enhance initial engagement for all learners (Koç and Savaş, 2025).

This study is not without limitations. The most critical limitation is the use of a single-item measure for English proficiency, which constrains the validity of the moderation analysis. Alongside the use of self-reported data and a sample from a single context, this suggests caution in generalizing the findings (Becker, 2005). Future research must prioritize longitudinal designs, incorporate multi-item, standardized test scores, or CEFR-aligned self-assessments to enhance reliability and enable more robust moderation analyses, and test the model in diverse settings and with different AI tool types. Investigating other potential moderators, such as digital literacy or learning agility, remains a vital direction (Liu et al., 2025).

In conclusion, this research provides initial evidence that while foundational skills like English proficiency remain important, they may not fundamentally alter the core acceptance pathways for well-designed AI learning tools in goal-oriented vocational settings. Pending verification with more robust measures, the value proposition of such technology appears capable of transcending traditional barriers, highlighting AI’s potential to democratize access to effective, personalized language education.

## Data Availability

The original data supporting the findings of this study are available within the article and/or Supplementary material. Where data cannot be shared due to ethical or privacy restrictions, they are available upon reasonable request from the corresponding author.
