# Supplementary material for: Does English proficiency matter? Testing its moderating role in the TAM for AI-enhanced MOOC adoption in vocational education
Source: Front Psychol. 2026 Feb 19;17:1772129. doi: 10.3389/fpsyg.2026.1772129 (PMC12960105; doi:10.3389/fpsyg.2026.1772129)
Supplement: Supplementary file 1 [file Supplementary_file_1.docx]

Appendix A The Survey Instrument

Part 1: Demographic and Background Information

| Variable | Item Wording (English) | Response Options |
| --- | --- | --- |
| Gender | Your gender: | Male / Female |
| Year of Study | Your current year of study: | Year 1 / Year 2 / Year 3 |
| English Proficiency | Before this course, how would you rate your overall English proficiency? | Beginner / Elementary / Intermediate / Upper-Intermediate / Advanced |

Part 2: Core Scales (retained items)

| **Construct** | **Item Code** | **Item Wording** | **Source (Adapted from)** |
| --- | --- | --- | --- |
| **Perceived Usefulness (PU)** | PU1 | Using AI-driven adaptive MOOC improves my English learning efficiency. | Davis (1989) |
|  | PU2 | The AI recommendations help me focus on knowledge gaps that need practice. |  |
|  | PU4 | The AI-driven system helps me achieve my learning goals more quickly. |  |
| **Perceived Ease of Use (PEOU)** | PEOU5 | The interface of the AI-driven MOOC platform is easy to navigate. |  |
|  | PEOU7 | The system provides clear guidance on how to use its features. |  |
|  | PEOU8 | I find the AI recommendations easy to understand and follow. |  |
| **Behavioral Intention (BI)** | BI9 | I intend to continue using AI-driven adaptive MOOCs in the future. | Venkatesh & Davis (2000) |
|  | BI10 | I will recommend this system to my peers. |  |
|  | BI12 | I am willing to prioritize using this type of AI-driven adaptive learning system in my future English studies. |  |
| **Perceived Learning Outcomes (PLO)** | PLO13 | The AI-driven adaptive learning system has significantly improved my English proficiency. | Wei, Saab, & Admiraal (2024) |
|  | PLO15 | The adaptive learning path effectively helped me master the key knowledge points. | Yuan & Liu (2025) |
|  | PLO16 | Using this system has boosted my confidence in learning English well. | Wei, Saab, & Admiraal (2024) |
|  | PLO17 | Overall, I am satisfied with the learning progress I achieved through this AI-enhanced MOOC. | Developed for this study |

Appendix B Results of Exploratory Factor Analysis (N=516)

| Construct | Item Code | Item Description (English) | Factor Loading | Communality |
| --- | --- | --- | --- | --- |
| **Perceived Usefulness (PU)** | PU1 | Using the AI-driven adaptive MOOC improved my English learning efficiency. | 0.852 | 0.726 |
|  | PU2 | The AI recommendation feature helped me focus on my knowledge weaknesses that needed practice. | 0.892 | 0.796 |
|  | PU4 | The AI-driven system helped me achieve my learning goals faster. | 0.882 | 0.778 |
| **Perceived Ease of Use (PEOU)** | PEOU5 | The interface of the AI-driven MOOC platform was easy to use. | 0.955 | 0.912 |
|  | PEOU7 | The system provided clear guidance on how to use its features. | 0.955 | 0.912 |
|  | PEOU8 | I found the AI-recommended content easy to understand and follow. | 0.930 | 0.865 |
| **Behavioral Intention (BI)** | BI9 | I intend to continue using AI-driven adaptive MOOCs in the future. | 0.937 | 0.878 |
|  | BI10 | I will recommend this system to my classmates. | 0.937 | 0.879 |
|  | BI12 | I am willing to prioritize using this type of AI-driven adaptive learning system in my future English studies. | 0.892 | 0.796 |
| **Perceived Learning Outcome (PLO)** | PLO13 | This AI-driven adaptive learning system significantly improved my English proficiency. | 0.902 | 0.813 |
|  | PLO15 | The adaptive learning path effectively helped me master the key knowledge points of the course. | 0.938 | 0.880 |
|  | PLO16 | Using this system has boosted my confidence in learning English well. | 0.922 | 0.849 |
|  | PLO17 | Overall, I am satisfied with the learning progress I achieved through this AI-enhanced MOOC. | 0.920 | 0.847 |

Notes:

1. KMO and Bartlett’s Test: All four constructs met the criteria for factor analysis (KMO values: PU = 0.723, PEOU = 0.760, BI = 0.739, PLO = 0.860; all Bartlett’s tests were significant at p < .001).

2. Variance Explained: A single factor was extracted for each construct, explaining a high percentage of total variance (PU: 76.70%, PEOU: 89.64%, BI: 85.12%, PLO: 84.75%).

3. Item Quality: All factor loadings and communality values exceed the recommended thresholds of 0.70 and 0.40, respectively, indicating excellent convergent validity and that the items are well-represented by their respective latent factors.

Appendix C Results of Harman’s Single-Factor Test

C1. Unrotated Variance Explained

| Component | Eigenvalue | % of Variance | Cumulative % |
| --- | --- | --- | --- |
| 1 | 7.601 | 58.468 | 58.468 |
| 2 | 0.877 | 6.743 | 65.211 |
| 3 | 0.613 | 4.713 | 69.924 |
| 4 | 0.548 | 4.219 | 74.143 |
| 5 | 0.482 | 3.706 | 77.849 |
| 6 | 0.451 | 3.467 | 81.316 |
| 7 | 0.418 | 3.216 | 84.532 |
| 8 | 0.407 | 3.132 | 87.663 |
| 9 | 0.352 | 2.708 | 90.371 |
| 10 | 0.337 | 2.596 | 92.967 |
| 11 | 0.320 | 2.463 | 95.430 |
| 12 | 0.313 | 2.405 | 97.835 |
| 13 | 0.281 | 2.165 | 100.000 |

Note: Results are based on an unrotated principal component analysis of all 13 measurement items, constrained to extract a single factor for Harman’s single-factor test (Podsakoff et al., 2003).

### C2. Unrotated Component Matrix (Loadings on the First Factor)

| Item | Unrotated Loading |
| --- | --- |
| PU1 | 0.757 |
| PU2 | 0.740 |
| PU4 | 0.755 |
| PEOU5 | 0.748 |
| PEOU7 | 0.728 |
| PEOU8 | 0.743 |
| BI9 | 0.780 |
| BI10 | 0.760 |
| BI12 | 0.808 |
| PLO13 | 0.787 |
| PLO15 | 0.794 |
| PLO16 | 0.747 |
| PLO17 | 0.788 |

Note: Loadings are from the unrotated principal component analysis. All items loaded on the first factor with loadings ≥ 0.728, consistent with the high inter-item correlations observed in the correlation matrix.

Appendix **D** Simple Slopes Calculation for Interaction Effects (H4–H6)

| **Figure & Hypothesis** | **b₁ (Predictor)** | **b₃ (Interaction)** | **High EP Slope** | **Low EP Slope** |
| --- | --- | --- | --- | --- |
| H4: PEOU × English → PU (Fig 2) | 0.749 | −0.040 | 0.631 | 0.697 |
| H5: PU × English → BI (Fig 2) | 0.695 | −0.003 | 0.686 | 0.691 |
| H6: BI × English → PLO (Fig 3) | 0.810 | −0.046 | 0.674 | 0.750 |

Note: English Proficiency (EP) mean = 2.13, SD = 0.82; High EP = M + 1SD = 2.95; Low EP = M – 1SD = 1.31. Calculated using Slope = b₁ + b₃ × EP value. See text for details.
